# Modulation of the Primary Astrocyte-Enriched Cultures’ Oxylipin Profiles Reduces Neurotoxicity

**DOI:** 10.3390/metabo11080498

**Published:** 2021-07-30

**Authors:** Mariia V. Guryleva, Dmitry V. Chistyakov, Alexander V. Lopachev, Sergei V. Goriainov, Alina A. Astakhova, Yulia A. Timoshina, Anastasiya V. Khutorova, Tatiana N. Fedorova, Marina G. Sergeeva

**Affiliations:** 1 Faculty of Bioengineering and Bioinformatics, Moscow Lomonosov State University, 119234 Moscow, Russia; guryleva.mv@gmail.com; 2 Belozersky Institute of Physico-Chemical Biology, Lomonosov Moscow State University, 119992 Moscow, Russia; alina_astakhova@yahoo.com (A.A.A.); mg.sergeeva@gmail.com (M.G.S.); 3 Laboratory of Clinical and Experimental Neurochemistry, Research Center of Neurology, 125367 Moscow, Russia; lopachev@neurology.ru (A.V.L.); july.timoschina@yandex.ru (Y.A.T.); hutorova.anastasiya@mail.ru (A.V.K.); tnf51@bk.ru (T.N.F.); 4 SREC PFUR, Peoples’ Friendship University of Russia (RUDN University), 117198 Moscow, Russia; goryainovs@list.ru; 5 Biological Department, Lomonosov Moscow State University, 119991 Moscow, Russia

**Keywords:** astrocytes, neurons, ML355, Zileuton, lipoxygenases, interleukin 10 (IL-10), interleukin 6 (IL-6), neuroinflammation, oxylipins, toll-like receptors (TLRs), neurotoxicity, ERK1/2

## Abstract

Recently, manipulations with reactive astrocytes have been viewed as a new therapeutic approach that will enable the development of treatments for acute brain injuries and neurodegenerative diseases. Astrocytes can release several substances, which may exert neurotoxic or neuroprotective effects, but the nature of these substances is still largely unknown. In the present work, we tested the hypothesis that these effects may be attributed to oxylipins, which are synthesized from n-3 or n-6 polyunsaturated fatty acids (PUFAs). We used astrocyte-enriched cultures and found that: (1) lipid fractions secreted by lipopolysaccharide (LPS)—stimulated rat primary astrocyte-enriched cultures—possessed neurotoxic activity in rat primary neuronal cultures; (2) both of the tested oxylipin synthesis inhibitors, ML355 and Zileuton, reduce the LPS-stimulated release of interleukin 6 (IL-6) by astrocyte cultures, but only ML355 can change lipid fractions from neurotoxic to non-toxic; and (3) oxylipin profiles, measured by ultra-performance liquid chromatography-tandem mass spectrometry (UPLC-MS/MS) from neurotoxic and non-toxic lipid fractions, reveal a group of n-3 docosahexaenoic acid derivatives, hydroxydocosahexaenoic acids (HdoHEs)-4-HdoHE, 8-HdoHE, and 17-HdoHE, which may reflect the neuroprotective features of lipid fractions. Regulating the composition of astrocyte oxylipin profiles may be suggested as an approach for regulation of neurotoxicity in inflammatory processes.

## 1. Introduction

Astrocytes are glial cells that provide trophic support for neurons and fulfill a substantial number of various homeostatic maintenance functions [1,2,3]. Currently, it is accepted that astrocytes play an active role in neuroinflammation [3]. Moreover, astrocytes are increasingly viewed as having a critical contribution to neurological disorders, including neurodegenerative and demyelinating diseases, epilepsy, trauma, ischemia, infection, and cancer [1,2]. Recently, manipulations with reactive astrocytes have been viewed as a new therapeutic approach, providing an opportunity to develop treatments for acute injuries and chronic diseases of the central nervous system (CNS) [1]. Neuroinflammation can be induced at the cellular level, and following various forms of stimulation, astrocytes undergo rapid changes in gene expression, morphology, and functions [1,2,3,4]. The functions of astrocytes depend on their contact with each other [5,6] and other cell types [7], the presence of proteins and the composition of the extracellular matrix [8,9,10], and even glucose concentrations in the medium [11,12,13]. An important problem in the use of brain cell cultures is their separation and manipulation during purification. The most commonly used culture is the so-called enriched astrocyte culture [14]. Although it contains a small amount of microglia, cells undergo minimal processing during isolation and culture [14]. Therefore, we used an astrocyte-enriched culture to study the effect of glial cells stimulated by inflammatory stimuli on neurons. It was shown that stimulation of microglia by LPS in microglia-astrocytes co-cultures [2] or direct activation of astrocytes by LPS [15] resulted in signals of unknown nature that induce neuron death by releasing toxic signal molecules [2]. Astrocytes can release various immune and inflammatory mediators, such as pro- and anti-inflammatory cytokines/chemokines and oxylipins, which may subsequently exert neurotoxic or neuroprotective effects [2,3,16]. The nature of the signals released by astrocytes is still questioned.

In the present work, we tested the hypothesis that astrocytes’ toxic message can be attributed to oxylipins. Oxylipins include various substances, which are synthesized from n-3 or n-6 PUFAs, such as arachidonic (AA, 20:4n-6), linoleic (LA, (18:2n-6), eicosapentaenoic (EPA, 20:5n-3), and docosahexaenoic (DHA, 22:6n-3) acids. The conversion of PUFAs into oxylipins occurs via three major pathways, involving cyclooxygenases (COX), lipoxygenases (LOX), and cytochrome P450 monooxygenases (CYP450) [17]. Oxylipins include both pro-inflammatory compounds and resolution substances, responsible for restoring the system after the pro-inflammatory stimulus has been applied [16,18]. Previously, the effects of individual substances were tested for their neuroprotective or neurotoxic activity [19,20,21,22]. Taken together, the data indicate that the study of individual substances may be useful in the search for potential therapeutic drugs; however, oxylipins must be studied in mixtures to understand the biology of inflammatory processes. Indeed, the activation of cells, caused by a pro-inflammatory stimulus, induces the simultaneous release of various types of oxylipins [15,23]. Although many oxylipins are released in low concentrations, their effects can be additive [24]. Therefore, the resulting response may be manifested as a combination of the mutual effects of individual oxylipins in experiments on cellular or organism levels. It follows that neuroprotective properties should be tested not for individual oxylipins but for their mixtures.

Recently we have shown that oxylipin profiles are associated with different astrocyte responses and reflect pro-inflammatory or anti-inflammatory phenotypes [15]. The pro-inflammatory phenotype was obtained via stimulation of astrocytes by LPS [15]. The LOX signaling pathway is of particular interest in the study of inflammation in the brain. It has been shown that enzymes of this pathway are involved in the neuropathogenesis and may be targets for the treatment of diseases such as stroke [25], Alzheimer’s disease [26], and other diseases [27].

In the present work, we compared lipid fractions from naive and LPS-stimulated primary astrocyte-enriched cultures in relation to their neurotoxic activities in the primary cortical neuron culture. To assess the possibility of modulating the neurotoxic activity of the fractions, we compared two substances, ML355 and Zileuton, which were added to the astrocytes, prior to LPS stimulation. ML355 and Zileuton belong to a class of LOX-mediated oxylipin synthesis inhibitors. The neuroprotective effects of the LOX pathway were discussed long ago [28,29], but precise mechanisms are still under research, mainly due to the uncertain view regarding the role of oxidative stress [30]. The inhibitors of LOX-mediated oxylipin synthesis manifest anti-inflammatory properties. For example, it was shown that Zileuton decreases brain damage and reduces inflammatory cytokines expression in the CNS [31,32]. Therefore, we estimated the possibilities of ML355 and Zileuton for the modulation profiles of oxylipin synthesis in astrocytes, indicating a further manifestation of lipid fractions’ neurotoxic activity.

## 2. Results

### 2.1. Lipid Fractions from LPS-Modulated Astrocytes Exerted Neurotoxicity That Can Be Modulated via Astrocytes’ Drug Treatments

**Figure 1:**
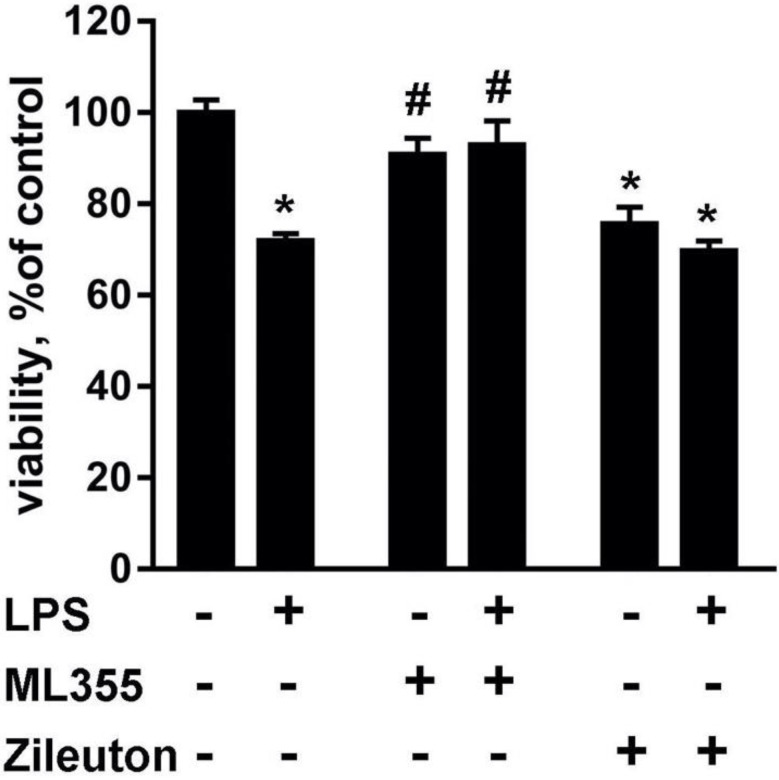
Lipid fraction from LPS-treated astrocytes changes the viability of neurons, which can be manipulated via astrocyte treatments with ML355 and Zileuton. The primary rat cortex neuron culture was incubated for 48 h with lipid fractions, which were prepared from an extracellular medium of astrocytes, activated for 24 h by LPS (100 ng/mL) only or with Zileuton (10 μM) or ML355 (ML 10 μM). Cell viability was measured using the MTT test. N = 12; *—*p* < 0.05, compared with the neurons treated with lipid fraction obtained from native astrocytes; #—*p* < 0.05, compared with the neurons treated with lipid fraction obtained from LPS-stimulated astrocytes.

It was shown previously that astrocyte supernatants, responding to inflammatory stimuli, induced neuronal death [2]. To assess the contribution of oxylipins to the observed toxic effects, we stimulated astrocytes with LPS (100 ng/mL, 24 h), then extracted the lipid fraction (see Materials and Methods). The fractions were added to cultured neurons, to a final amount of 1% of the total medium volume. An MTT test was then conducted, and its results were compared to the parameters of the control neuron culture, which was incubated with 1% solution for isolating lipid fractions (Figure 1). Lipid fractions secreted by LPS-treated astrocytes possessed neurotoxic activity and caused a 28 ± 1.5% (*p* < 0.001) decrease in primary rat cortex neuron culture viability (Figure 1, left pair of columns). For modulation, we added ML355 or Zileuton to the astrocyte cultures for 30 min, then incubated the cells with and without LPS for 24 h. Lipid fractions after Zileuton treatment were toxic, both from naive and LPS-treated astrocytes (Figure 1). Zileuton-treated or Zileuton plus LPS-treated astrocyte fractions yielded a 24.3 ± 3.6% (*p* < 0.001) or 30.3 ± 2.2% (*p* < 0.001) decrease in viability, respectively, in comparison to the control culture. The fractions from ML355-treated astrocytes did not influence neuron culture viability (*p* = 0.64). Moreover, the addition of ML355 removed the toxic effect of LPS. Neuron cultures, treated with the LPS and ML355, displayed a 21 ± 5.2% higher vitality than those treated with LPS (*p* = 0.001). Therefore, modulation of oxylipin synthesis by ML355 eliminates the toxic effect of lipid fraction.

### 2.2. The Toxic Effect Is Accompanied by the Activation of ERK1/2 in Neurons

**Figure 2:**
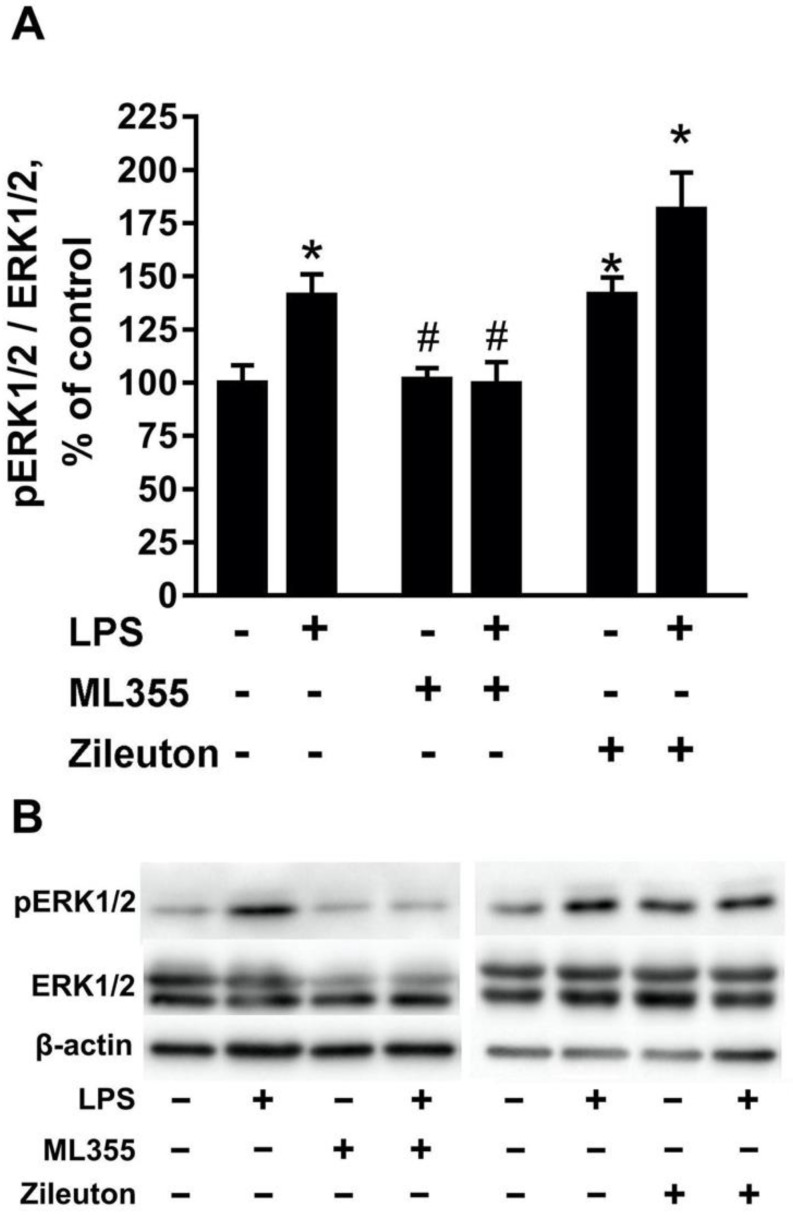
Effects of lipid fractions from the culture medium of treated astrocytes on ERK1/2 activity in neuronal cultures. The lipid fractions from astrocytes treated with LPS, ML355, LPS + ML355, Zileuton, and LPS + Zileuton were added to the primary rat cortex neuron cultures for 4 h. pERK1/2 and ERK1/2 protein levels were evaluated by Western blotting and normalized to the loading control β-actin. (**A**) Results, expressed as fold-changes, relative to the control. (**B**) Representative Western blots demonstrating phospho-ERK1/2, total ERK1/2, and β-actin protein levels. The example is representative of four independent experiments. The values represent mean ± SD from four independent experiments. *—*p* < 0.05, compared with the neurons treated with lipid fraction obtained from native astrocytes; #—*p* < 0.05, compared with the neurons treated with lipid fraction obtained from LPS-stimulated astrocytes.

The activation of the mitogen-activated protein kinase ERK1/2 in neurons is known to play an important role in the manifestation of neurotoxicity [33]. Therefore, we compared the levels of ERK1/2 phosphorylation after treatments with tested lipid fractions. For this purpose, primary rat cortex neuron cultures incubated with tested lipid fractions for 4 h, then protein lysates were analyzed using Western blotting (Figure 2). The lipid fractions of the astrocytes treated with LPS, Zileuton, or Zileuton + LPS displayed increased levels of ERK1/2 activity in neurons (Figure 2). The lipid fractions of astrocytes treated with ML355 or in combination with ML355 + LPS did not affect the levels of ERK1/2 activity (Figure 2). Therefore, the neurotoxicity of lipid fractions is associated with increased levels of ERK1/2 activity.

### 2.3. LPS-Induced Release of Cytokines Is Modulated by Zileuton and ML355

**Figure 3:**
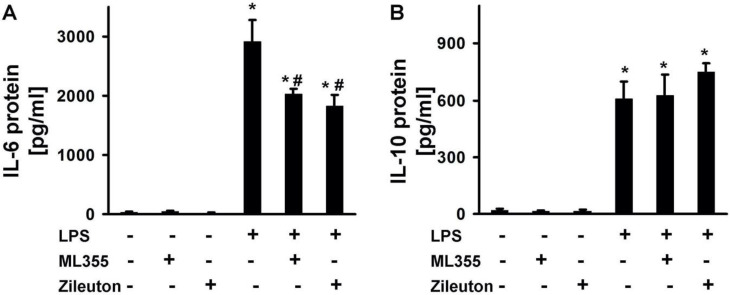
A comparison of ML355 and Zileuton effects on the LPS-induced release of interleukin 6 (IL-6) and interleukin 10 (IL-10) in astrocytes. The primary rat astrocyte cultures were pretreated with Zileuton (10 μM) or ML355 (10 μM) for 30 min and then stimulated with LPS (100 ng/mL) for 24 h. The IL-6 (**A**) and IL-10 (**B**) protein release was measured by ELISA in the supernatant samples. The results are expressed as pg/mg. The values represent a mean ± SD from three independent experiments. * *p* < 0.05, compared with the non-stimulated cells; # *p* < 0.05, compared with the LPS-stimulated cells.

Previously, it has been shown that the addition of LOX-inhibitors modulated levels of pro-inflammatory (IL-6, TNFα) and anti-inflammatory (IL-10) cytokines [31,34]. In order to compare the effects of ML355 and Zileuton on the LPS-induced release of cytokines in astrocytes, we evaluated the release of IL-6 and IL-10 on the protein level (Figure 3). There was no difference between the tested substances, ML355 or Zileuton; both decreased the levels of LPS-stimulated IL-6 release (Figure 3A). The tested substances do not modulate this release of IL-10 from astrocytes, stimulated with LPS (Figure 3B). Thus, both substances decreased the level of LPS-induced cytokine IL-6 release, and therefore they likely worked in astrocyte-enriched cultures, but there is no difference between them in the tested astrocytes’ function of cytokine releases.

### 2.4. The Difference in the Neurotoxicity of Lipid Fractions Is Reflected in Their Oxylipin Profiles

**Figure 4:**
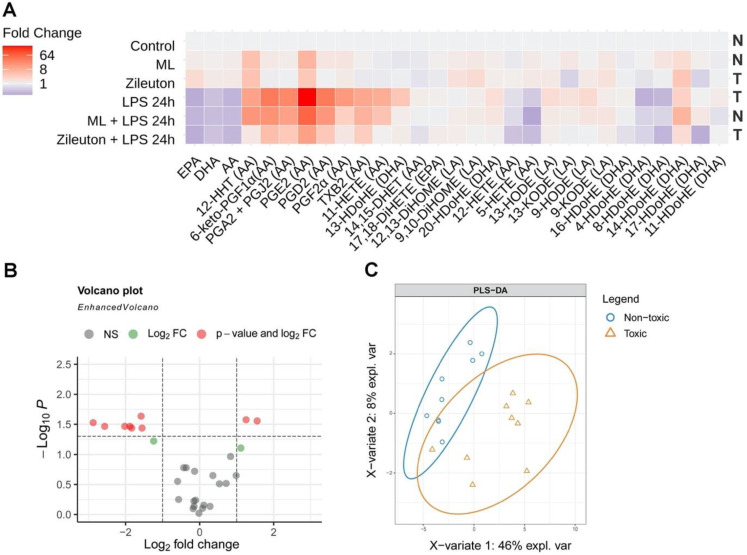
ML355 and Zileuton modulate oxylipin profiles of the LPS-stimulated astrocytes. The primary rat astrocyte cultures were pretreated with Zileuton (10 μM) or ML355 (10 μM) for 30 min and then stimulated with LPS (100 ng/mL) for 24 h. The concentrations of oxylipins in the supernatants were measured using ultra-performance liquid chromatography-tandem mass spectrometry (UPLC-MS/MS). (**A**) The heat map shows the relative amounts of each lipid mediator, compared to the control. The vertical axis indicates the stimuli, while the horizontal axis indicates each lipid mediator’s relative amount (log2), N—non-toxic fraction and T—toxic fraction. (**B**) The volcano plot indicates significantly changed compounds. The *X*-axis indicates a log2 fold change of toxic fractions. The *Y*-axis indicates log10 *p*-values (adjusted). The cut-off for *p*-values is indicated, based on Bonferroni correction. Compounds that changed insignificantly are indicated in gray, and compounds, the means of which changed in toxic fractions (relative to neuroprotective fractions) more than twofold or less than twofold but insignificantly, are indicated in green. Red dots stand for compounds which changed more than twofold and had a *p*-value (adjusted < 0.05). (**C**) The partial least square discriminant analysis (PLS-DA) model discriminates toxic (T) and non-toxic (N) for neuron fractions. The explained variance of each component is indicated in brackets on the corresponding axis.

To identify the difference between lipid fractions, we analyzed them using the approach we developed previously with the mass-spectrometric method of oxylipin profiles detection [35,36]. We obtained oxylipin profiles from astrocytes treated solely with LPS, ML355, and Zileuton or in combinations for 24 h (Figure 4A). We identified 29 compounds, among them three PUFAs (DHA, EPA, and AA) and derivatives of COX, cytochrome P450 monooxygenases (CYP), and LOX metabolic pathways. Data are presented as a heatmap, in which the vertical axis indicates the stimuli, while the horizontal axis indicates the relative amount (log2) of each lipid mediator (Figure 4A) (quantitative data presented in Table S1).

For further analysis, we subdivided the lipid fractions, according to the data shown in Figure 1, into “toxic” (LPS, Zileuton, Zileuton + LPS) and “non-toxic” (control, ML355, LPS + ML355). To evaluate the individual metabolites that differ between the non-toxic and toxic groups, we performed pairwise comparisons of metabolite concentrations. The results were then illustrated using a volcano plot with Holm–Bonferroni correction (Figure 4B). The two metabolites, the concentrations of which were significantly increased (4-HdoHE, 8-HdoHE), and the seven metabolites, the concentrations of which were significantly decreased (13-HdoHE, PGE2, PGA2 + PGJ2, PGD2, PGF2a, 11-HETE, 6-keto-PGF1a), are indicated in red (Table 1).

To test whether toxic and non-toxic fractions could be distinguished based on oxylipin concentrations, partial least square discriminant analysis (PLS-DA) was performed. The projections relating to the first two components are presented in Figure 4C. The tested fractions were separated with a small overlap. For each metabolite, the VIP score was estimated. The value of this parameter addresses the explained variation between classes in each projection. Three metabolites, including 13-HdoHE, 4-HdoHE, and 17-HdoHE, with VIP score values >1.5, are shown in Table 2. The PLS-DA revealed three oxylipins, which are derivatives of DHA, as potential substances of neuroprotective signals from the lipid fractions of astrocytes for neurons.

## 3. Discussion

Intercellular signaling in response to pro-inflammatory stimuli is being actively studied. Understanding these signals would both shed light on inflammatory processes as a whole and would assist the search for new therapeutic approaches. Glia–neuron interactions are key to understanding various CNS dysfunctions, including neurodegenerative diseases, since inflammation is a significant factor in the development of these pathologies. As a rule, astrocytes promote CNS neuronal survival [37]. However, pro-inflammatory, activated astrocytes induce neuronal apoptosis via release of a toxic signal [2]. Our study suggests new targets in this area of research, and points to the potential involvement of oxylipins in this process. We found that (1) lipid fractions obtained from the extracellular medium of LPS-stimulated primary astrocyte-enriched cultures possessed neurotoxic properties; (2) their action can attenuated via treatment of astrocytes with ML355, but not Zileuton; (3) the decrease of viability of neuron cultures is accompanied by an increased level of ERK1/2 activity; (4) both ML355 and Zileuton cause a decrease in the LPS-stimulated release of IL-6, but not IL-10, in astrocyte-enriched cultures, indicating the activity of these substances in these cultures; and (5) the comparison of oxylipin profiles from neurotoxic and non-toxic lipid fractions reveals a group of DHA derivatives, which may reflect non-toxic features of lipid fractions.

Our data indicating that the lipid fractions derived from the extracellular medium of LPS-stimulated astrocytes possessed neurotoxic activity concur with previously published data, in which a toxic soluble signal from so-called A1 polarized astrocytes has been shown [2]. The authors suggested that substances targeting this signal could constitute new drugs, with great potential for treating a variety of chronic, neurological diseases [2]. We hypothesized that the toxic signal might, at least in part, be associated with the lipid fraction released by stimulated astrocytes. Our data are in accordance with this suggestion. For estimating the possibility of reducing neurotoxicity through the modulation of oxylipins released by stimulated astrocytes, we used inhibitors of the LOX pathway. Previously, the neuroprotective properties of LOX inhibitors were evaluated in experiments, where the inhibitors were added directly to the neurons [28,29]. In our work, we tested two inhibitors, ML355 (an inhibitor of 12-LOX) and Zileuton (an inhibitor of 5-LOX) [38,39]. ML355 is a selective inhibitor with favorable absorption, distribution, metabolism, and excretion (ADME) properties [40]. We did not find data on the effect of ML355 on astrocytes. The drug was suggested for antiplatelet therapy and diabetes [38,40]. Our data reveal that this substance can also potentially be used to reduce the neurotoxicity of astrocytes in inflammatory processes. At present, it is difficult to say if this is a unique property of ML355, or if other inhibitors of 12-LOX can act in a similar way. Another tested drug, Zileuton, is a selective inhibitor of 5-LOX, which is a key enzyme in the biosynthesis of the leukotrienes and has been implicated in central nervous system (CNS) disorders such as Alzheimer’s disease and acute ischemic stroke [31,32]. Our data on the modulation of cytokines by zileuton were in accordance with previously published work [34], where it was shown on human astrocytes that the 5-LOX-inhibitor zileuton and the 5-, 12-, and 15-LOX-inhibitor 2-TEDC significantly reduced the expression of multiple pro-inflammatory cytokines. Therefore, both tested inhibitors are active in LPS-stimulated astrocyte-enriched culture, but their effects on released oxylipins differ in view of the neuroprotective features of oxylipin profiles.

At present, the mechanisms of the inhibitor actions are not obvious. For example, 14-HdoHE, the DHA metabolites, can be produced by 15-LOX in neutrophils [41] or 12-LOX in platelets [42]. If one attributes HDoHEs to LOX activities in astrocytes, there is still the question of why the addition of LOX inhibitors induced LOX-mediated derivatives. The observed differences may reflect the indirect action of ML355 and Zileuton since we used the long-treated times of the cells; therefore, different feedback mechanisms could manifest themselves. Mechanisms of ML355 and Zileuton modulation of oxylipin synthesis seem to be more complicated on a cellular level [39,43]. Our data cannot be allowed to discriminate molecular mechanisms of LOX inhibitors’ effects in astrocytes but reveal an opportunity to manipulate the neurotoxic effects of LPS-treated astrocyte-enriched cultures.

The question remains, what is the desired oxylipin profile? There is currently little knowledge of the complex nature of oxylipins’ metabolism at a cellular level. Indeed, pro-inflammatory signals cause the activation of various phospholipases A2, and the released PUFAs are distributed between the different branches of metabolism (COX, LOX, P450) [17]. In addition, there is a non-enzymatic pathway for the oxidation of PUFAs, which depends on the redox status of cells [44]. Only recently, with the development of mass spectrometric methods for the simultaneous detection of many oxylipins, has it become possible to study the system of PUFA metabolism and the synthesis of oxylipins at the level of cellular responses [45,46]. The approach we used to compare different lipid fractions in terms of their neurotoxicity (we used Volcano plot and PLSA-DA) made it possible to determine that 10 compounds differ between the non-toxic fraction and the toxic fraction—13-HDoHE, 4-HdoHE, 8-HDoHE, PGE2, PGA2 + PGJ2, PGD2, PGF2a, 11-HETE, 6-keto-PGF1a, and 17-HdoHE. Among them, a group of substances that are usually attributed to the COX-metabolizing branch (13-HDoHE, PGE2, PGA2 + PGJ2, PGD2, PGF2a, 11-HETE, and 6-keto-PGF1a) was decreased.

If we try to characterize the actions of oxylipin mixtures according to the existing data on individual oxylipins, we come across a paucity of studies. The data are contradictory even in regard to such a well-known substance as PGE2. The bifunctional effects of PGE2 on inflammatory conditions have been documented. While high concentrations of PGE2 (5–25 μM) induced apoptosis in cultured rat hippocampal cells [19], low concentrations of PGE2 (0.01 to 10 nM) protected dopaminergic neurons against LPS-induced neurotoxicity in rat primary neuron-glial cultures [20]. It was shown that the effect was mediated by the modulation of the microglial part of the mixed culture [20]; therefore, an indirect effect of PGE2 was assumed. The existence of negative feedback effects of PGE2 or PGD2 that down-regulate other pro-inflammatory reactions is known [16,21]. Our data reveal the effect of oxylipins in other situations, as we add oxylipins directly to neuron cultures in “native” concentrations extracted directly from the medium of astrocyte-enriched treated cultures. We can only note that it seems that PGE2 and PGD2 possess neurotoxic effects because their concentrations were decreased in mixtures with neuroprotective features.

Both AA and DHA derivatives were found in the fraction which possessed neuroprotective properties. The substances 17-HdoHE, 4-HdoHE, and 8-HdoHE were increased. All of these are derivatives of DHA and may be attributed to a neuroprotective oxylipin fraction. This is a logical assumption for several reasons. Five major oxygenated metabolites, derived from DHA, were identified in the rat brain: 17-HDoHE, 14-HDoHE, 11-HDoHE, 7-HDoHE, and 4-HDoHE [47]. It is known that the substances 4-HDoHE, 14-HDoHE, and 17-HDoHE belong to a group of specialized, pro-resolving mediators (SPMs); they are also the precursors of other SPMs [18,48]. Moreover, they previously were suggested as SPM biosynthetic pathway markers [49]: for example, 17-HDoHE for the formation of RvD, and protectin D1 and 14-HDoHE for the biosynthesis of maresin 1 [48]. Maresin, RvD, and protectin D1 promote inflammatory resolution, neuroprotection, and functional neurological recovery following brain injury [48,50]. Therefore, the protective properties of lipid fractions enriched with these substances do not seem unusual.

Taken together, our data support previous research findings that astrocytes, activated by pro-inflammatory stimuli, release soluble signals, which exert neurotoxic effects. We have shown for the first time that these neurotoxic effects are mediated, at least in part, via oxylipin mixtures. Each oxylipin is synthesized individually in small amounts, but their effects can be additive. Regulation of the oxylipin mixture composition results in changes in the cellular responses. We have demonstrated the possibility of such modulation using the ML355 oxylipin synthesis inhibitor. The use of selective inhibitors of the synthesis of oxylipins makes it possible to modulate the mixture of oxylipins and thereby reduce neuroinflammation. This approach can have positive therapeutic consequences in treating diseases with an inflammatory component such as stroke, Alzheimer’s disease, and Parkinson’s disease.

## 4. Materials and Methods

### 4.1. Reagents

Lipopolysaccharide (cat.no. L2630) was from Sigma-Aldrich, St. Louis, MO, USA, and ML355 (cat.no. 18537) and zileuton (cat.no. 10006967) were from Cayman Chemical, Ann Arbor, MI, USA. Streptomycin–penicillin (cat.no. A063), trypsin (cat.no. P037), EDTA, and fetal bovine serum (cat.no BS-110/500) were from PanEco, Moscow, Russia. Culture medium Dulbecco’s Modified Eagle Medium (DMEM) (cat.no. 21885-025) was from Gibco, Thermo Fisher Scientific, Waltham, MA, USA. Antibodies against p-ERK1/2 (Thr202/Tyr204, cat.no. sc-136521) and ERK1/2 (cat.no. sc-135900) were from Santa Cruz Biotechnology, Dallas, TX, USA. β-Actin (cat.no. 8457) and anti-mouse IgG-HRP (cat.no. 7076) were from Cell Signaling Technology, Danvers, MA, USA. Membranes were developed using Western blotting substrate ECL (Thermo Fisher Scientific, cat.no 32209, Waltham, MA, USA). ELISA kits for IL-10 (cat.no. 555134) and IL-6 (cat.no. 550319) were from BD Biosciences, San Diego, USA, San Diego, CA, USA. The oxylipins standards were as follows: 6-keto PGF1α-d4 (cat.no. 315210), TXB2-d4 (cat.no. 319030), PGF2α-d4 (cat.no. 316010), PGE2-d4 (cat.no. 314010), PGD2-d4 (cat.no. 312010), 5(S)-HETE-d8 (cat.no. 334230), 12(S)-HETE-d8 (cat.no. 334570), 15(S)-HETE-d8 (cat.no. 334720), EPA-d5 (cat.no, 10005056), DHA-d5 (cat.no. 10005057), and AA-d8 (cat. No. 390010) were from Cayman Chemical, Ann Arbor, MI, USA. Oasis^®^ PRIME HLB cartridges (60 mg, 3cc, cat.no. 186008056) were obtained from Waters, Eschborn, Germany.

### 4.2. Primary Rat Astroglial-Enriched Cell Culture

Astrocyte-enriched cultures were obtained from newborn rats of both sexes as previously reported [11,51]. More than 95% of the cells were positive for the astrocyte marker glial fibrillary acidic protein in these cultures, and only <2% were positive for a microglia-specific marker. In brief, brains from newborn rats were triturated by the use of nylon meshes of 250 and 136 μm pore width, in consecutive order. Dissociated cells were plated into 75 cm^2^ culture flasks at a density of 6 × 10^5^ cells per ml. The cells were subsequently cultured in DMEM (1 g/L D-glucose, 10% bovine fetal serum (FBS), 50 units/mL streptomycin, 50 μg/mL penicillin) at 37 °C, with 10% CO_2_. After 5 days of cultivation in DMEM, the culture medium was replaced with a fresh medium, and flasks were placed on a shaker at 200 RPM for 4 h to dissociate microglial cells. The culture medium was changed to a fresh one. After 2 days, the monolayer of astrocytes was trypsinized and plated into six-well plates and maintained for 2 days in DMEM. After this, the medium was changed, and the cells were used for experiments. All of the experimental procedures were performed according to the guidelines in the European Convention for the Protection of Vertebrate Animals used for Experimental and Other Scientific Purposes. MTT assay was used to determine changes in viability following exposure of confluent astrocyte-enriched cultures to ML355 and Zileuton and other pre- and co-treatments. No loss of viability occurred in astrocyte-enriched cultures (data not shown).

### 4.3. Primary Cultures of Rat Neuron Cortical Cells

To obtain the cultures, cerebral hemispheres were isolated from Wistar rat embryos on day 18 of gestation. The procedure was carried out as has been described earlier [52]. Afterwards, cultures were maintained in Neurobasal Medium (NBM) (Gibco, Thermo Fisher Scientific, 168 Third Avenue, Waltham, MA, USA) with 2% B-27 Serum Free Supplement (Gibco), 100 U/mL penicillin-streptomycin, and 1% GlutaMAX (Gibco) in the incubator (SHEL LAB, Cornelius, OR, USA) at 37 °C, 90% humidity, 5% CO_2_ for 10–12 days. Half of the medium volume was refreshed every 2 days.

### 4.4. MTT Assay

Cell viability of the culture was evaluated in 96-well plates using MTT test. The method is based on the reduction of yellow 3-(4,5-dimethyl-2-thiazolyl)-2,5-diphenyl-2H-tetrazolium bromide (MTT) by living cells to blue formazan. Water-insoluble crystals on the bottom of the wells were dissolved in 100 µL per well DMSO (AppliChem, Maryland Heights, MO, USA). Sample absorbance at 570 nm and 660 nm was measured using a Synergy H1 plate reader (BioTek, Winooski, VT, USA). Then, the absorbance value at 660 nm was subtracted from absorbance value at 570 nm. Data are presented as a percentage of the value in control wells with intact cells.

### 4.5. UPLC-MS/MS Conditions and Sample Preparation 

After the cell experiments, the supernatant was collected and stored at −80 °C for further analysis. The cell-free culture media were taken for the solid-phase lipid extraction (Oasis^®^PRIME HLB cartridge (60 mg, 3 cc)) and analyzed using 8040 series UPLC-MS/MS mass spectrometer (Shimadzu, Kyoto, Japan) in multiple-reaction monitoring mode as described previously [35]. The selected lipids were identified and quantified using Lipid Mediator Version 2 software (Shimadzu, Japan).

### 4.6. Western Blotting

ERK1/2 activation was evaluated using Western blotting. The procedure was carried out in the same manner as described in our earlier work [52]. Briefly, cultured cells in the 6-well plates were lysed in RIPA buffer (Sigma, Ronkonkoma, NY, USA) containing cocktails of protease and phosphatase inhibitors (Sigma, Ronkonkoma, NY, USA). The protein concentration was measured using DC Protein Assay Kit (Bio-Rad, Hercules, CA, USA). Luminescence was detected by means of ChemiDoc XRS+ system (Bio-Rad, Hercules, CA, USA), and the luminescence intensity was calculated with Image Lab 3.0 software (Bio-Rad, Hercules, CA, USA).

### 4.7. Data Analysis

Comparison of the relative oxylipin concentrations in two groups (toxic over non-toxic) was performed using the two-sample two-sided *t*-test. Benjamini–Hochberg correction was applied to control false discovery rate (FDR). *p* < 0.05 was considered as statistically significant.

Metabolomics data were analyzed using the mixOmics R package version 6.1.1 [53]. Class separation was analyzed by partial least square discriminant analysis (PLS-DA). The quality of the models with different number of components was estimated using leave-one-out cross-validation. In each round of cross-validation, training set included the bigger data subset, while validation was performed on a randomly selected sample. Predictive performance of models was compared based on overall error rate and AUC.

After building the PLS-DA model with 3 components, the relative importance of each metabolite was calculated using a parameter called the variable importance in projection (VIP). VIP score is a weighted sum of squares of the PLS loadings regarding the explained variation in each projection. A cutoff for VIP scores was accepted as 1.5 according to the metabolomics standard initiative (level MSI = 1).

## Figures and Tables

**Table 1:** Log2 FCs (fold changes) are shown for nine metabolites, *p* < 0.05 (adjusted for multiple testing).

Name	13-HDoHE	4-HDoHE	8-HDoHE	PGE2	PGA2 + PGJ2	PGD2	PGF2a	11-HETE	6-keto-PGF1a
log2FC	−1.58	1.25	1.56	−2.87	−1.88	−2.02	−2.56	−1.55	−1.84

**Table 2:** Variable importance in projection (VIP) scores are shown for three metabolites. A cutoff value of 1.5 is established for VIP selection.

Name	13-HDoHE *	4-HDoHE *	17-HDoHE
VIP-scores	1.6235235	1.5420719	1.588623

* Volcano plot indicating significantly changed compounds, *p* < 0.05 (adjusted for multiple testing).

## Data Availability

Data are contained within the article or Supplementary Material.

## Supplementary Materials

The following are available online at https://www.mdpi.com/article/10.3390/metabo11080498/s1, Table S1: Mean (SD) PUFAs and oxylipins released from astrocytes treated with ML355 and Zileuton alone or in combination with LPS for 24 h fold to control.
