# Supplementary material for: Modulation of the Primary Astrocyte-Enriched Cultures’ Oxylipin Profiles Reduces Neurotoxicity
Source: Metabolites. 2021 Jul 30;11(8):498. doi: 10.3390/metabo11080498 (PMC8399552; doi:10.3390/metabo11080498)
Supplement: Supplementary file 1 [file metabolites-11-00498-s001.zip › metabolites-1305966-supplementary.pdf]

Supplementary: Modulation of the primary astrocyte-enriched cultures oxylipin profiles reduces neurotoxicity

Maria V. Guruleva [1], Dmitry V. Chistyakov [2], Alexander V. Lopachev [3], Sergei V. Goriainov [4], Alina A. Astakhova [2], Yulia A. Timoshina [3,5], Anastasiya V. Khutorova [3,5], Tatiana N. Fedorova [3], and Marina G. Sergeeva [2]

[1] Faculty of Bioengineering and Bioinformatics, Moscow Lomonosov State University, Moscow 119234, Russia; guryleva.mv@gmail.com
[2] Belozersky Institute of Physico-Chemical Biology, Lomonosov Moscow State University, Moscow 119992, Russia; alina_astakhova@yahoo.com, mg.sergeeva@gmail.com
[3] Laboratory of Clinical and Experimental neurochemistry, Research Center of Neurology, Moscow 125367, Russia; lopachev@neurology.ru, tnf51@bk.ru
[4] SREC PFUR Peoples' Friendship University of Russia (RUDN University), Moscow 117198, Russia; goryainovs@list.ru
[5] Biological Department, Lomonosov Moscow State University, 119991 Moscow, Russia; july.timoschina@yandex.ru, hutorova.anastasiya@mail.ru
* Correspondence: Chistyakof@gmail.com; Tel.: +74-95-939-4332

Table S1. Mean (SD) PUFAs and oxylipins released from astrocytes treated with ML355 and Zileuton alone or in combination with LPS for 24h fold to control.

No	LPS 24h	ML355	ML355 + LPS 24h	Zileuton	Zileuton + LPS 24h
Sample	mean ± SD	mean ± SD	mean ± SD	mean ± SD	mean ± SD
EPA	0.21±0.04	1.21±0.34	0.27±0.06	2.02±0.39	0.22±0.03
DHA	0.35±0.04	1.10±0.02	0.31±0.01	1.28±0.39	0.35±0.02
AA	0.26±0.03	1.20±0.27	0.30±0.04	1.34±0.41	0.28±0.01
12-HHT	11.29±4.06	6.71±0.20	16.65±5.44	4.36±1.05	1.43±1.44
6-keto-PGF1a	35.34±3.57	1.31±0.23	18.48±2.10	0.90±0.16	4.86±0.22
PGA2+PGJ2	24.45±2.12	1.21±0.43	11.77±2.46	0.95±0.23	3.52±0.22
PGE2	249.89±54.64	6.53±2.89	61.85±14.20	3.68±1.03	11.94±1.60
PGD2	36.13±5.35	1.47±0.45	16.01±2.20	0.98±0.20	3.68±0.64

PGF2a	15.09±6.65	1.50±0.20	2.84±0.80	1.28±0.31	1.44±0.47
TXB2	9.74±0.93	1.09±0.13	7.12±0.24	0.84±0.21	3.04±0.12
11-HETE	8.50±0.50	0.86±0.22	4.27±0.75	0.85±0.23	1.06±0.17
13-HDoHE	3.85±0.21	0.78±0.12	1.17±0.40	0.92±0.11	1.20±0.34
14.15-DHET	1.19±0.26	0.82±0.39	0.94±0.28	0.74±0.28	0.68±0.16
17.18-DiHETE	1.12±0.07	1.09±0.16	0.94±0.05	0.83±0.37	1.23±0.02
12.13-DiHOME	0.93±0.27	1.24±0.43	1.22±0.39	1.70±0.26	1.16±0.02
9.10-DiHOME	1.17±0.58	1.37±0.81	1.28±0.27	2.01±0.60	1.04±0.01
20-HDoHE	1.78±0.44	1.17±0.19	1.47±0.14	1.18±0.19	1.09±0.24
12-HETE	0.65±0.19	0.84±0.07	0.62±0.14	1.11±0.14	0.36±0.08
5-HETE	0.43±0.36	1.28±0.47	0.19±0.21	1.31±0.21	0.31±0.13
13-HODE	1.94±1.43	1.06±0.34	1.32±0.20	1.31±0.20	1.18±0.01
13-KODE	1.62±0.65	1.43±0.52	1.20±0.45	0.51±0.18	0.93±0.09
9-HODE	2.09±2.42	1.05±0.33	1.52±0.26	1.30±0.11	0.99±0.21
9-KODE	1.17±0.56	1.37±0.63	1.52±0.11	1.61±0.20	1.21±0.12
16-HDoHE	1.12±0.16	0.87±0.10	0.91±0.21	0.91±0.17	0.68±0.12
4-HDoHE	0.24±0.12	1.06±0.35	0.52±0.06	0.73±0.13	0.53±0.35
8-HDoHE	0.28±0.18	1.51±0.51	0.54±0.18	0.91±0.17	0.27±0.16

14-HDoHE	2.06±1.32	2.87±0.75	6.67±1.27	3.81±1.04	1.94±0.03
17-HDoHE	1.05±0.53	1.13±0.28	1.39±0.36	0.51±0.11	0.23±0.04
11-HDoHE	1.24±0.45	0.89±0.36	0.70±0.21	0.95±0.13	1.03±0.07
